# Supplementary material for: The origin of ultrahigh piezoelectricity in relaxor-ferroelectric solid solution crystals
Source: Nat Commun. 2016 Dec 19;7:13807. doi: 10.1038/ncomms13807 (PMC5187463; doi:10.1038/ncomms13807)
Supplement: Supplementary Information — Supplementary Figures, Supplementary Tables, Supplementary Notes and Supplementary References. [file ncomms13807-s1.pdf]

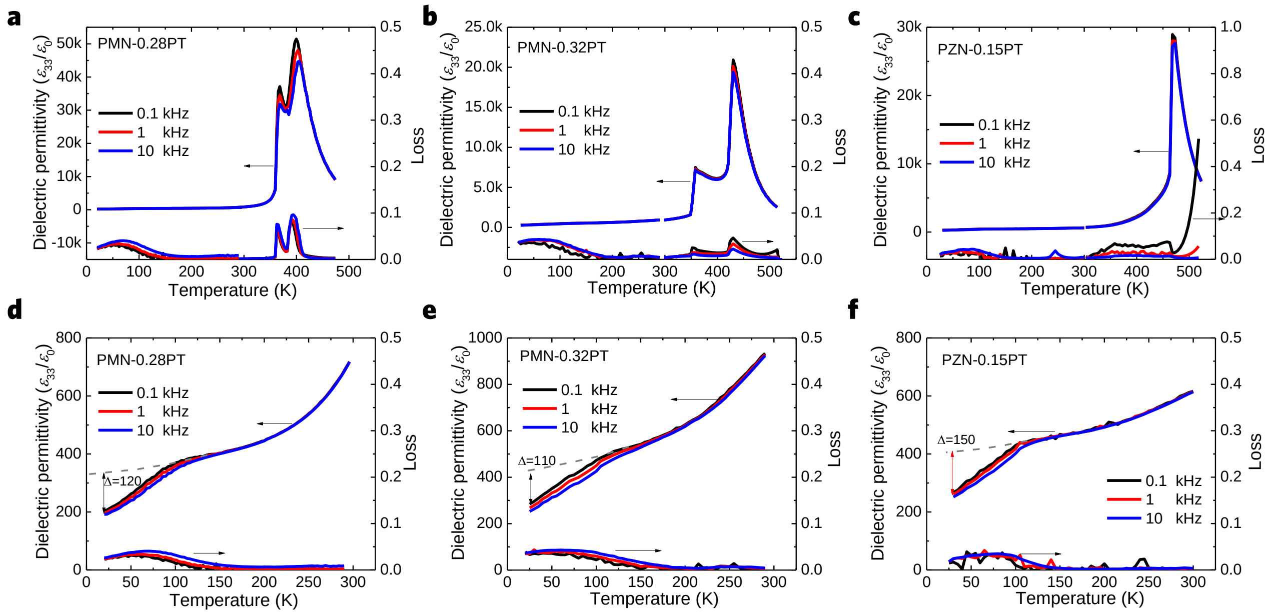

Supplementary Figure 1. Temperature dependence of the longitudinal dielectric permittivity for relaxor-PT crystals. **a**, rhombohedral PMN-0.28PT; **b**, orthorhombic PMN-0.32PT, and **c**, tetragonal PZN-0.15PT crystals. Figs. **d**, **e** and **f** are enlarged low temperature parts for Figs. **a**, **b** and **c**, respectively.

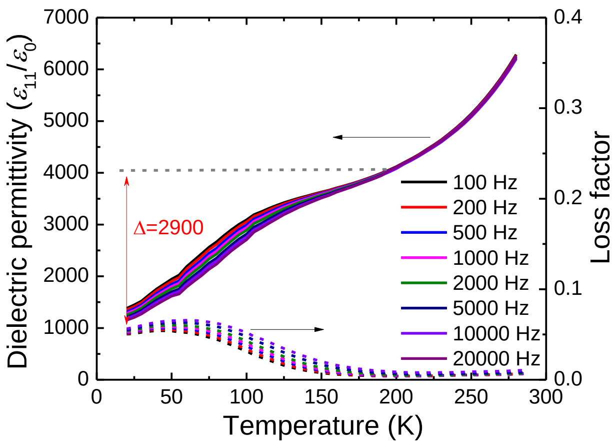

Supplementary Figure 2. The temperature dependence of the transverse dielectric permittivity $\varepsilon_{11}/\varepsilon_0$ for PMN-0.32PT crystals.

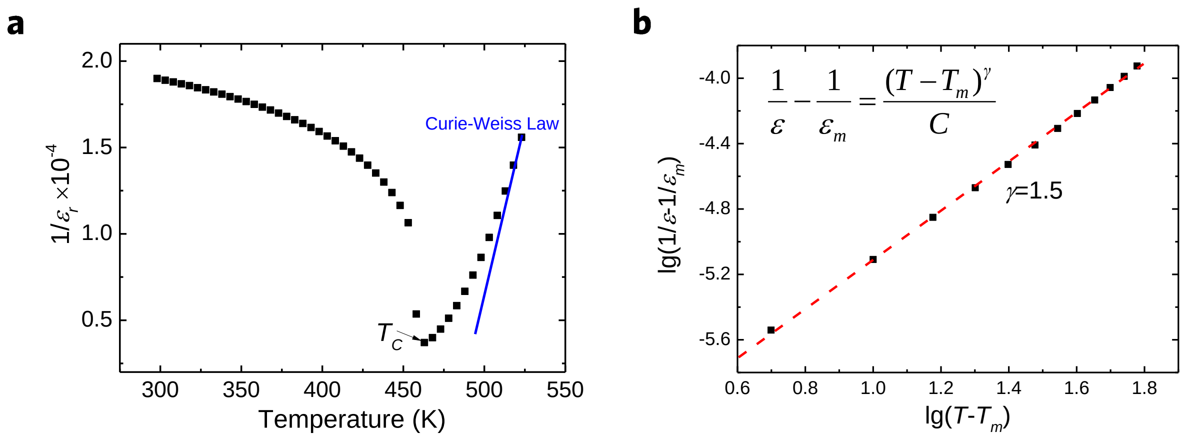

Supplementary Figure 3. Relaxor characteristic of the PZN-0.15PT crystal. **a**, Temperature dependence of the reciprocal of relative dielectric constant for a [001]-oriented PZN-0.15PT crystal; **b,** $\lg(1/\varepsilon-1/\varepsilon_m)$ vs. $\lg(T-T_m)$ figure at temperatures above T_m for obtaining parameter γ. γ is the degree of diffuseness. A higher value of γ represents a higher level of relaxor behavior. For classical ferroelectrics, the value of γ is 1, while for relaxor, the value is 2. The measured γ value of the PZN-0.15PT crystal is 1.5.

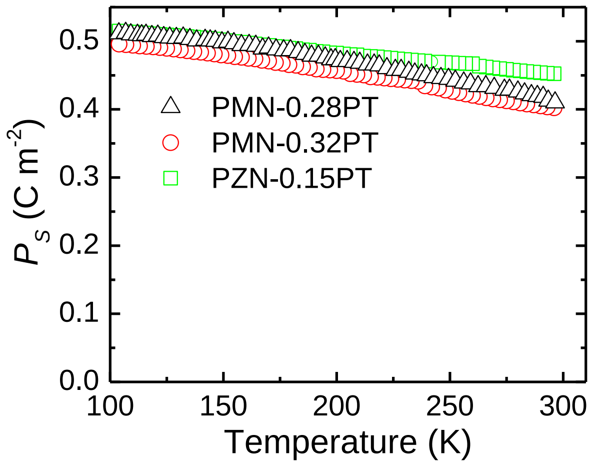

Supplementary Figure 4. The spontaneous polarization with respect to temperature for the PMN-0.28PT, PMN-0.32PT and PZN-0.15PT crystals. All the crystals were poled along their respective polar directions.

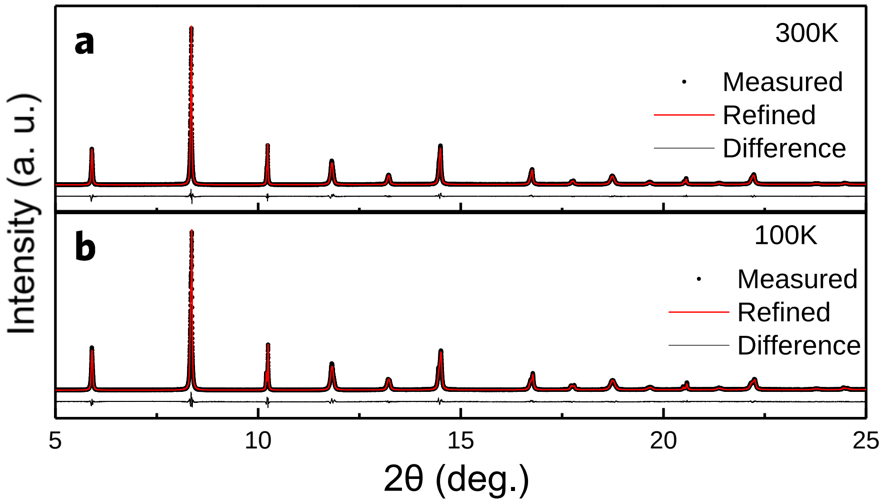

Supplementary Figure 5. Synchrotron powder XRD patterns for the PMN-0.28PT. **a**, at 300 K; **b**, at 100 K.

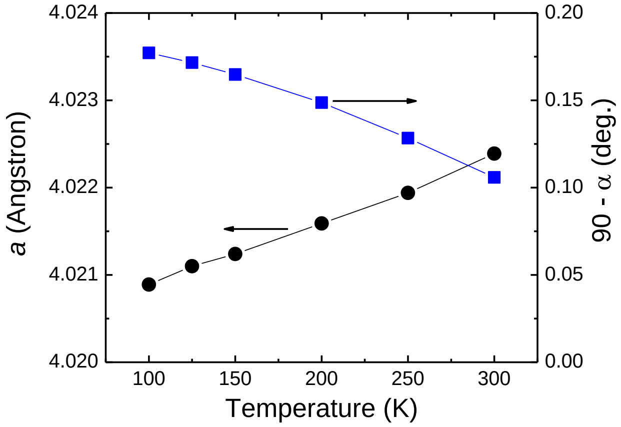

Supplementary Figure 6. Temperature-dependent lattice parameters for the PMN-0.28PT.

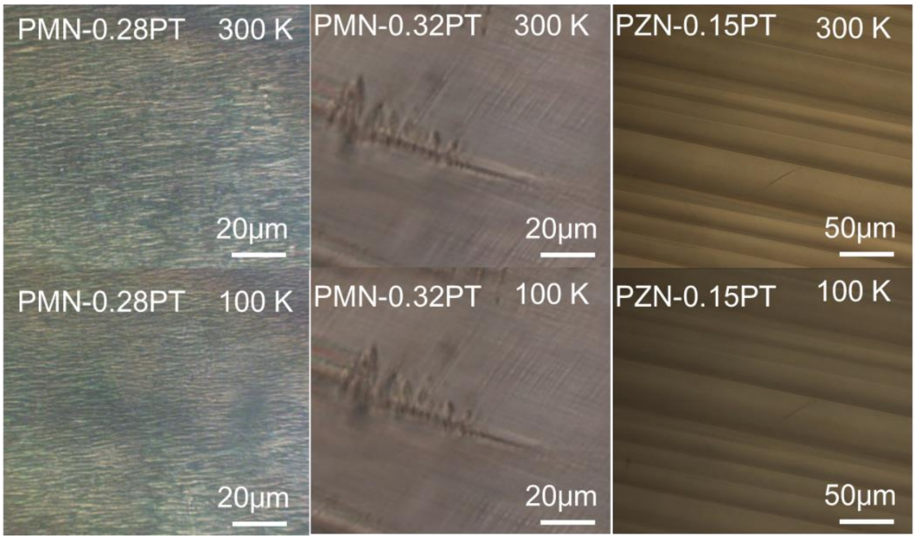

Supplementary Figure 7. Polarized light microscopic pictures for [001] oriented PMN-0.28, PMN-0.32 and PZN-0.15PT crystals at temperatures of 100 K and 300 K. The domain structures of relaxor-PT crystals were observed using a polarizing light microscope (PLM) (Olympus BX51) with a LINKAM heating-cooling stage. The samples were polished to 50-70 μm and annealed at 573 K for domain observations.

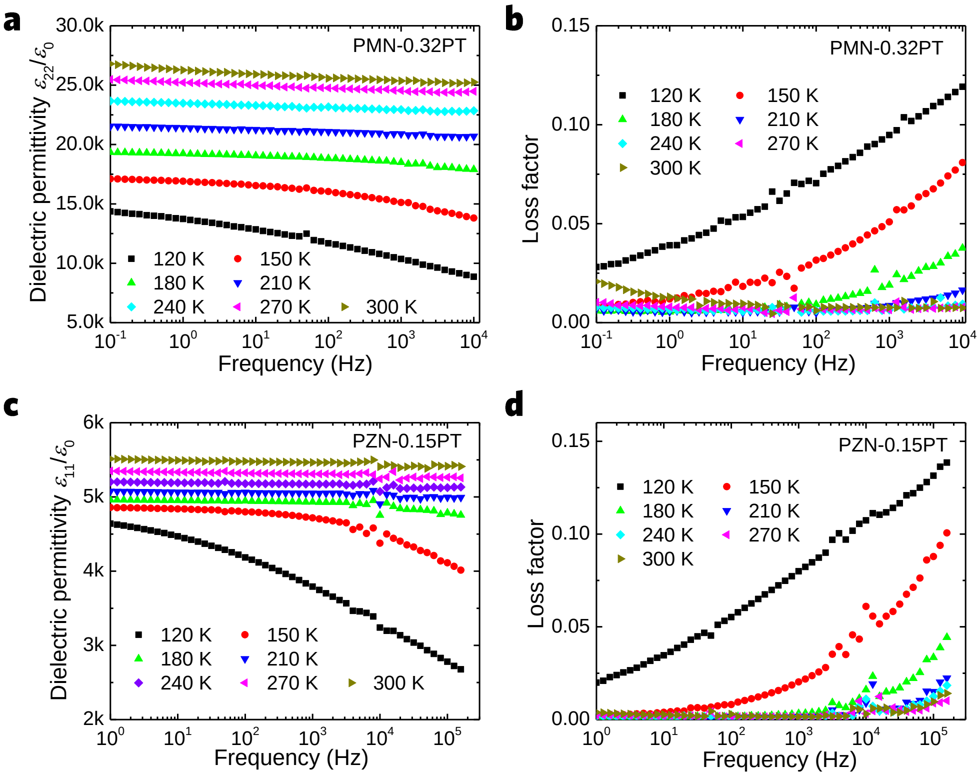

Supplementary Figure 8. Frequency spectra of transverse dielectric permittivity and loss factor for relaxor-PT crystals. **a**, dielectric permittivity of PMN-0.32PT; **b**, loss factor of PMN-0.32PT; **c**, dielectric permittivity of PZN-0.15PT; **d**, loss factor of PZN-0.15PT.

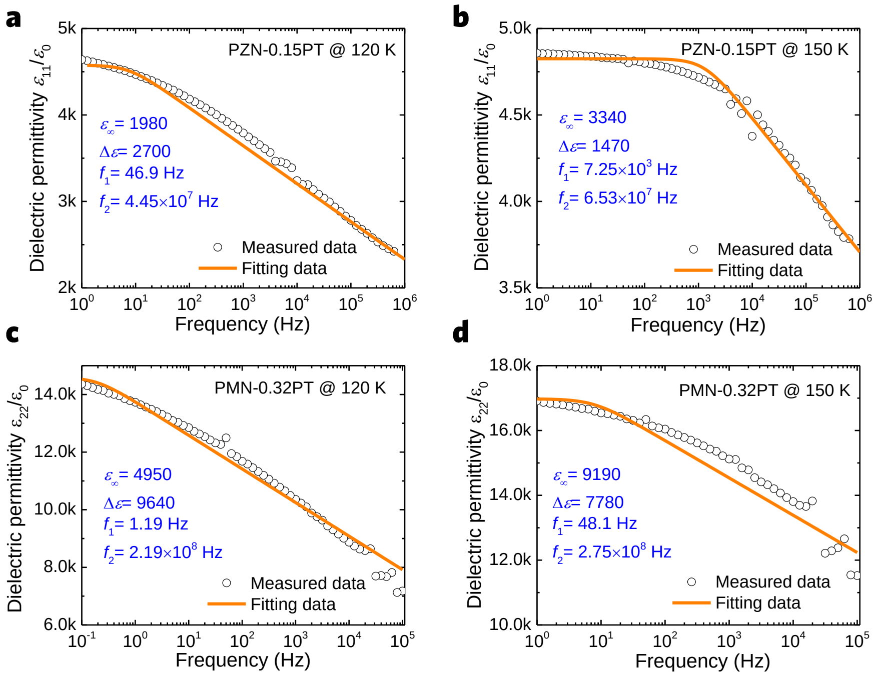

Supplementary Figure 9. Measured and fitted transverse dielectric permittivity vs. frequency for PMN-0.32PT and PZN-0.15PT crystals. **a**, PZN-0.15PT at 120 K; **b**, PZN-0.15PT at 150 K; **c**, PMN-0.32PT at 120 K; **d**, PMN-0.32PT at 150 K.

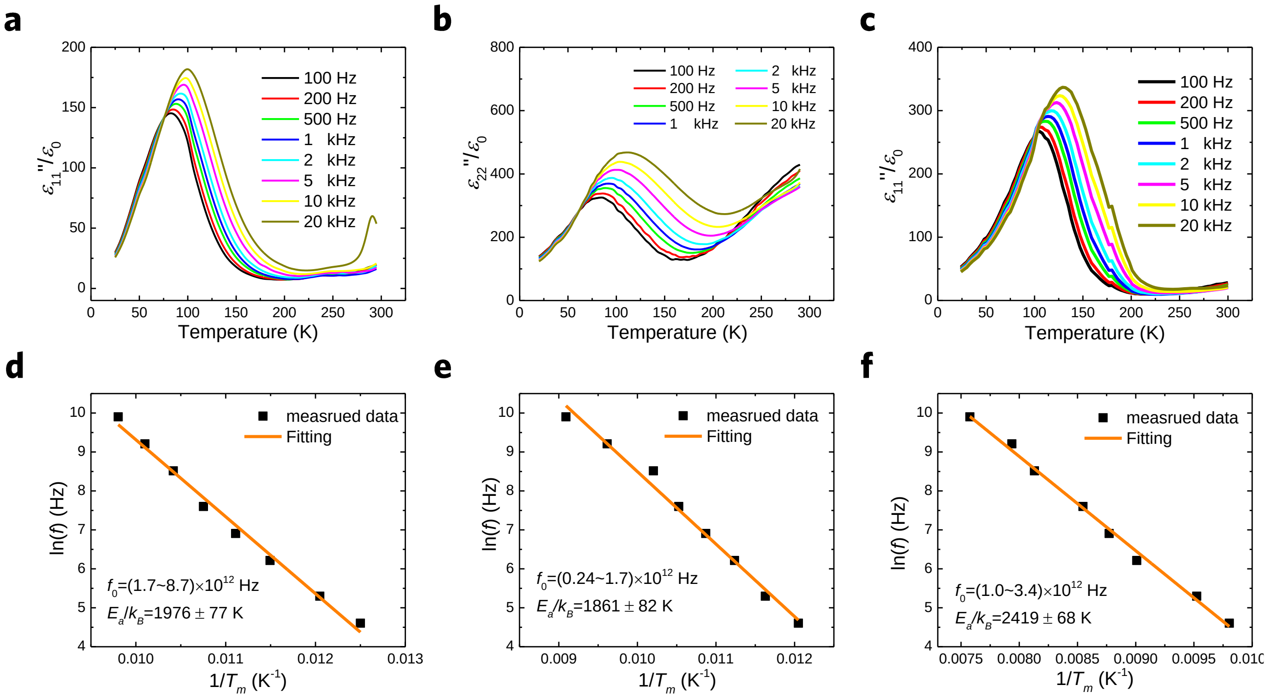

Supplementary Figure 10. Inference of the size of PNRs by Arrhenius law for relaxor-PT crystals. **a**, **b**, and **c** are the temperature dependence of the imaginary part ($\varepsilon''/\varepsilon_0$) of the transverse dielectric permittivity for PMN-0.28PT, PMN-0.32PT, and PZN-0.15PT crystals, respectively. **d**, **e**, and **f** are the T_ms (temperature of maximum $\varepsilon''/\varepsilon_0$) versus frequency for PMN-0.28PT, PMN-0.32PT and PZN-0.15PT crystals, respectively.

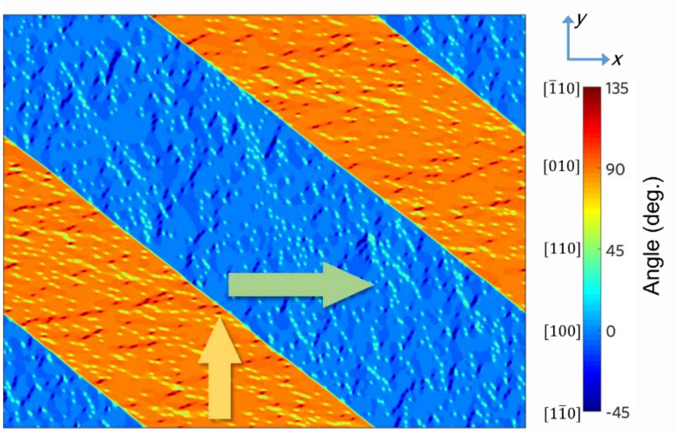

Supplementary Figure 11. Microstructure of a [110]-poled PNR-ferroelectric composite at 150 K (poled at room temperature, i.e., 300 K), where the *x*- and *y*-axes represent the [100] and [010] directions respectively. The scale of this simulation is 512×512 nm. The color bar denotes the angle between the polar vector and the [100] direction. Selected orientations are given in the color bar. The average polar directions of the macro-domains are represented by yellow and green arrows.

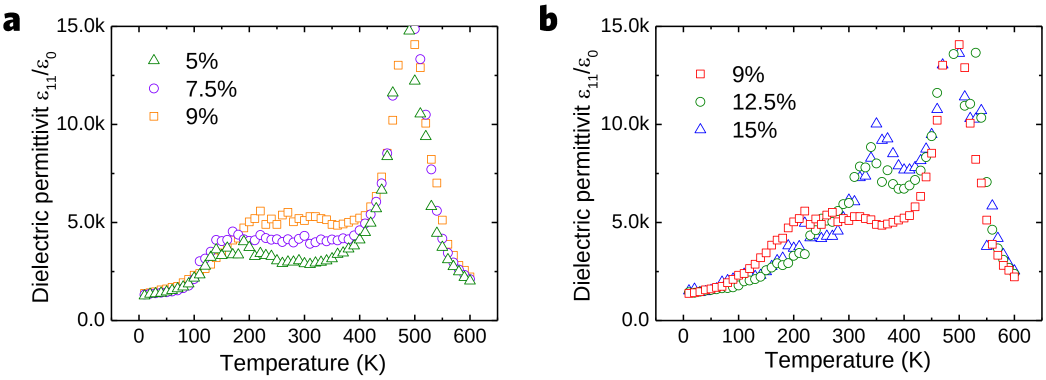

Supplementary Figure 12. Simulated transverse dielectric permittivity $\varepsilon_{11}/\varepsilon_0$ versus temperature for the [100]-poled PNR-ferroelectric composites at different volume fractions of the PNRs. **a**, volume fractions of 5%, 7.5% and 9%; **b**, volume fractions of 9%, 12.5% and 15%.

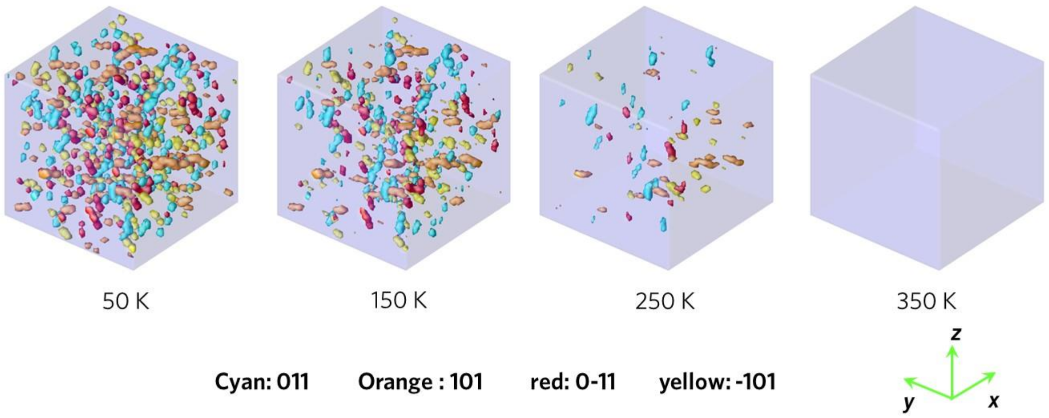

Supplementary Figure 13. Microstructure of a [001]-poled PNR-ferroelectric composite with respect to temperature. The translucent-blue color represent the polar vectors along [001] direction. The cyan, orange, red and yellow colors represent the polar vector along [011], [101], [0$\bar{1}$1] and [$\bar{1}$01] directions, respectively. Actually, the exactly tetragonal- and orthorhombic-type polar vectors will not exist due to the interaction between ferroelectric matrix and PNRs. In this figure, the angle deviation of 10° is allowed for plotting different color regions. The *x*-, *y*- and *z*-axes represent the [100], [010] and [001] directions, respectively.

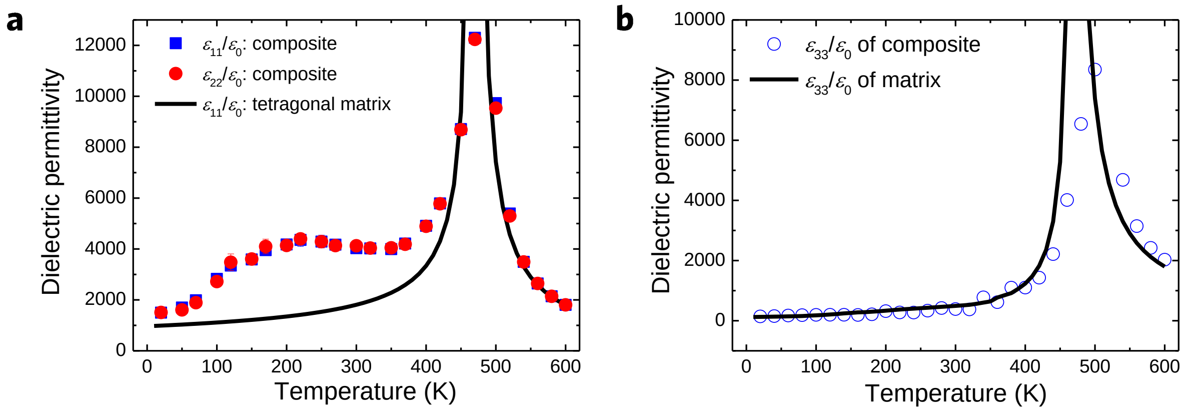

Supplementary Figure 14. Simulated dielectric permittivites versus temperature for a [001]-poled PNR-ferroelectric composite. **a**, transverse dielectric permittivies; **b**, longitudinal dielectric permittivities. The dielectric permittivities $\varepsilon_{11}/\varepsilon_0$, $\varepsilon_{22}/\varepsilon_0$, and $\varepsilon_{33}/\varepsilon_0$ are measured along [100], [010] and [001] direction, respectively.

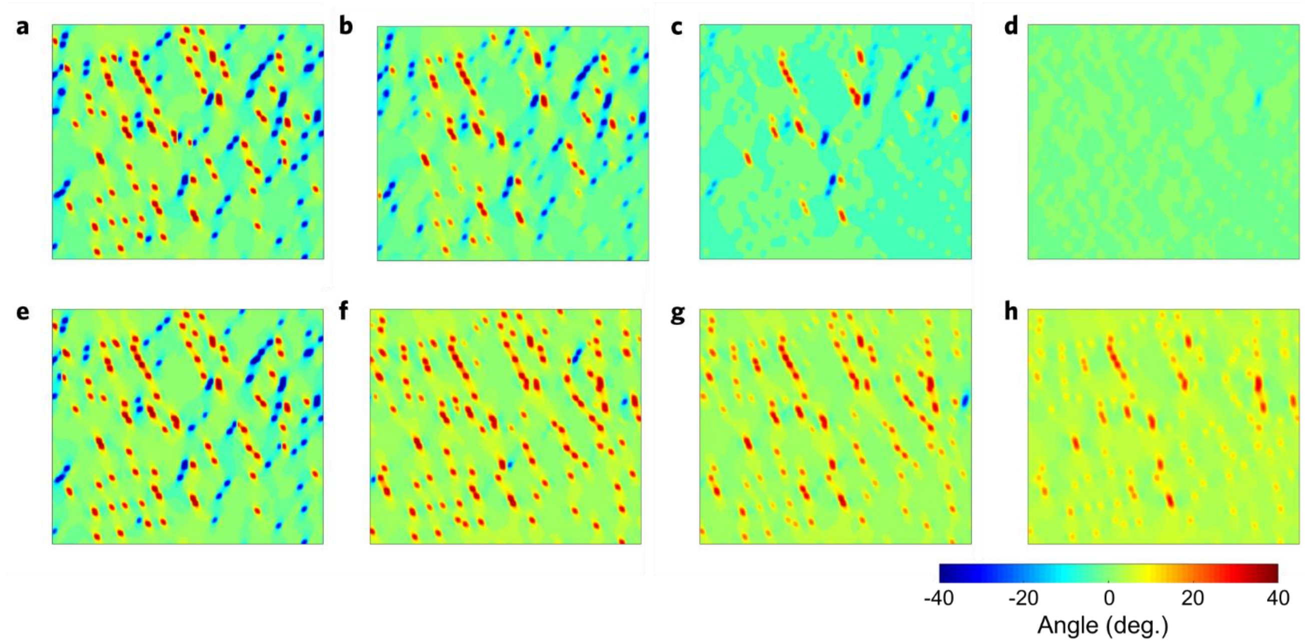

Supplementary Figure 15. Microstructure evolution of a [100]-poled PNR-ferroelectric composite under shear stress σ_{12}. The *x*- and *y*-axes represent [100] and [010] directions, respectively. The color bar denotes the angle (unit: degree) between the polar vector and the [100] direction. **a**, **b**, **c** and **d** are the microstructures under zero stress at 50 K, 150 K, 250 K and 350 K, respectively. **e**, **f**, **g** and **h** are the microstructures under 35 MPa at 50 K, 150 K, 250 K and 350 K, respectively.

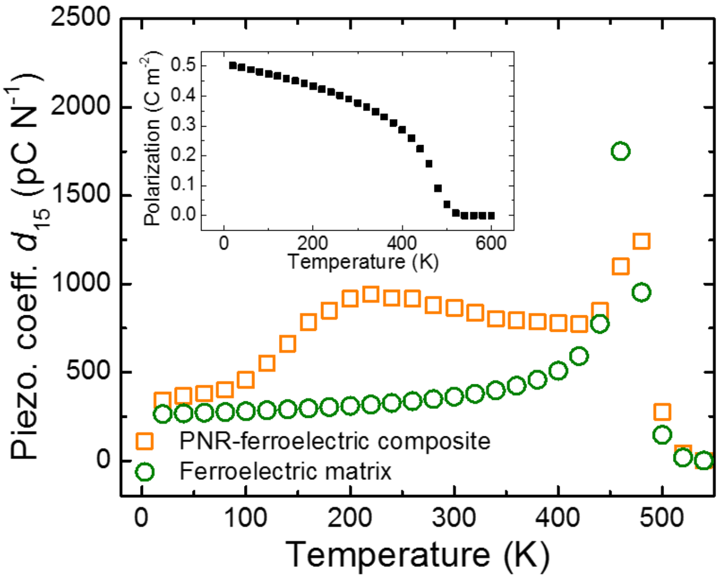

Supplementary Figure 16. The calculated temperature dependence of shear piezoelectric coefficient d_{15} for a PNR-ferroelectric composite and ferroelectric matrix. The amplitude and period of the applied stress is 10 MPa and 10^5 time steps, respectively. Compared with dielectric response, piezoelectric response show a slight decrease in the temperature range of 200 K-400 K. This is due to the decrease of spontaneous polarization with increasing temperature, as shown in the inset of this figure.

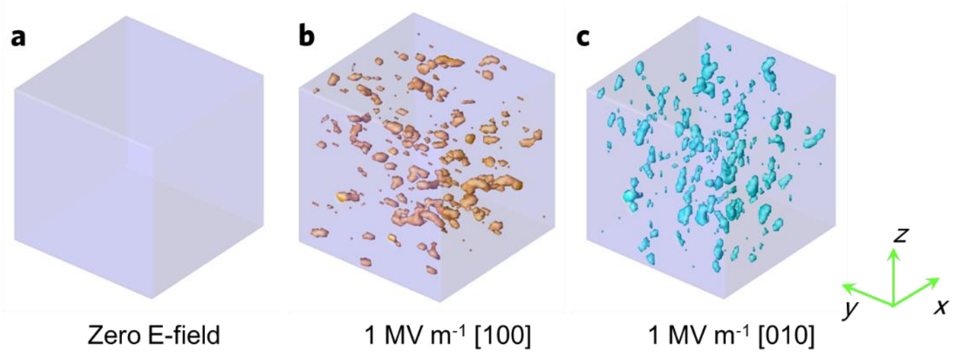

Supplementary Figure 17. The E-field induced microstructure variation for a [001]-poled PNR-ferroelectric composite at 350 K. **a**, under zero E-field; **b**, under a 1 MV m^{-1} E-field along the [100] direction; **c**, under a 1 MV m^{-1} E-field along the [010] direction. The translucent-blue color represents the polar vectors along [001] direction. The cyan and orange colors represent the polar vector along the [011] and [101] directions, respectively. The *x*-, *y*- and *z*-axes represent the [100], [010] and [001] directions, respectively.

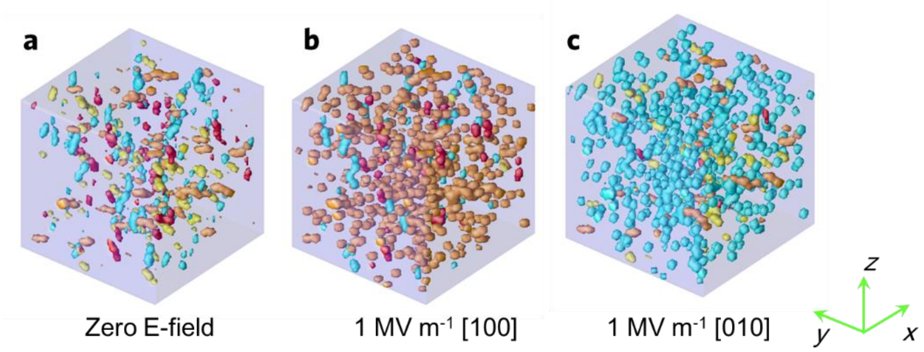

Supplementary Figure 18. E-field induced microstructure variation for a [001]-poled PNR-ferroelectric composite at 150 K. **a**, under zero E-field; **b**, under a 1 MV m^{-1} E-field along the [100] direction; **c**, under a 1 MV m^{-1} E-field along the [010] direction. The translucent-blue color represents the polar vectors along [001] direction. The cyan, orange, red and yellow colors represent the polar vector along the [011], [101], [0$\bar{1}$1] and [$\bar{1}$01] directions, respectively. The *x*-, *y*- and *z*-axes represent the [100], [010] and [001] directions, respectively.

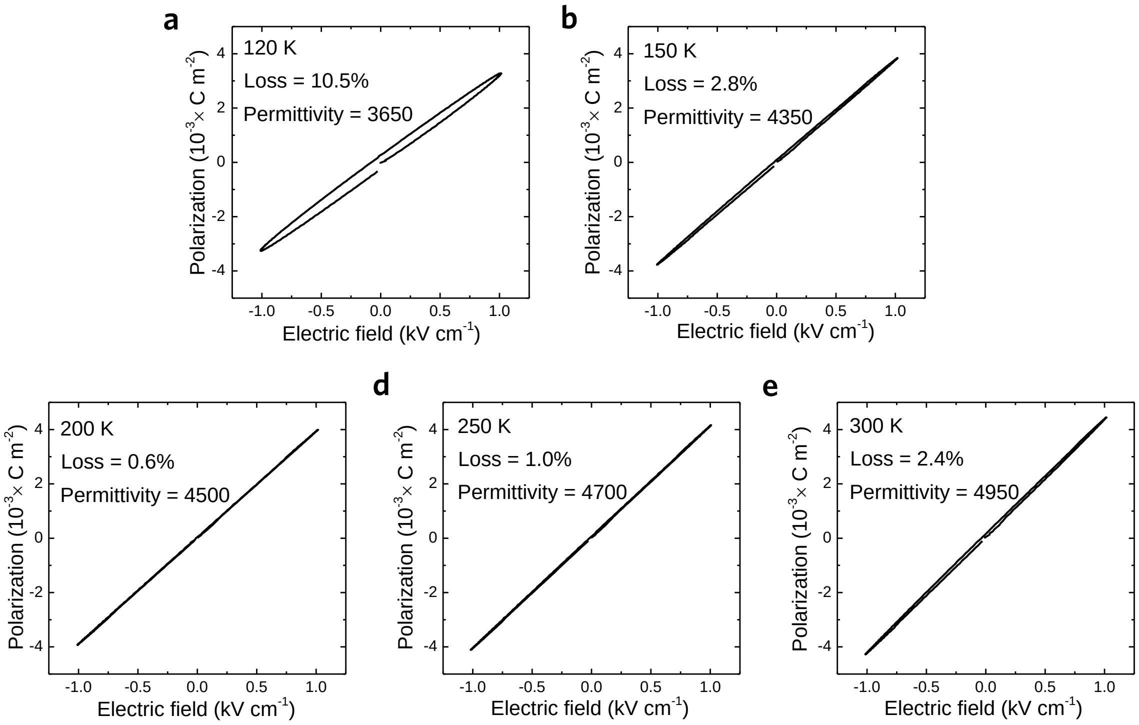

Supplementary Figure 19. Transverse polarization-electric field (PE) responses for PZN-0.15PT crystals at various temperatures. **a**, 120 K; **b**, 150 K; **c**, 200 K; **d**, 250 K; **e**, 300 K. The test frequency is 100 Hz.

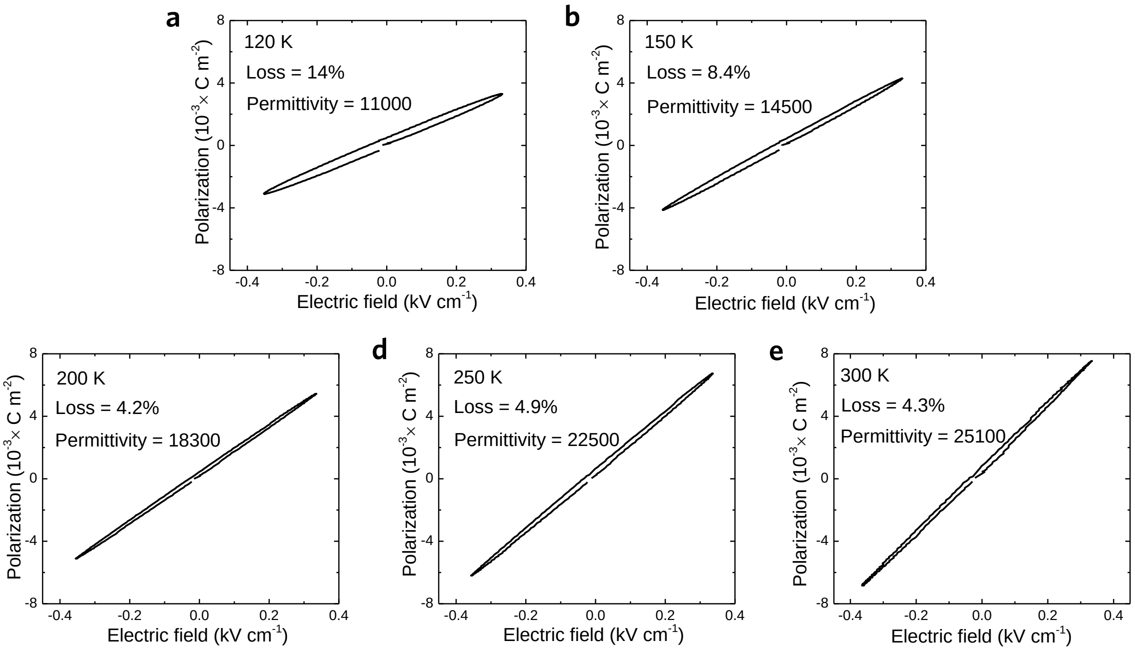

Supplementary Figure 20. Transverse PE responses (22-mode) for PMN-0.32PT crystals at various temperatures. **a**, 120 K; **b**, 150 K; **c**, 200 K; **d**, 250 K; **e**, 300 K. The test frequency is 100 Hz.

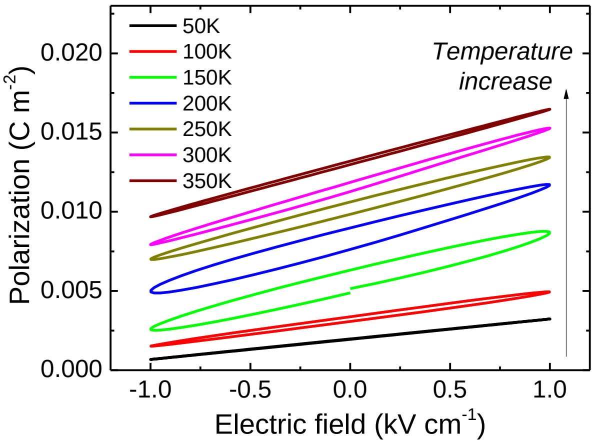

Supplementary Figure 21. Simulated PE of transverse dielectric response for the PNR-ferroelectric composite. The driving field is 1 kV cm^{-1}. The reduced period of ac electric field is 10^5 time steps. The simulated results are similar to the measured one, as shown in Supplementary Figures 19-20.

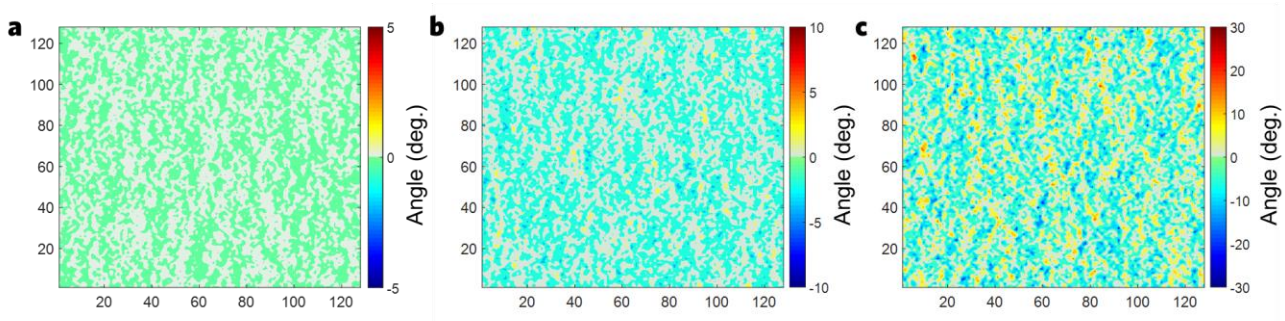

Supplementary Figure 22. Simulated domain morphology for the [100]-poled tetragonal matrix at different magnitudes of thermal noise. **a**, **b**, and **c** represent the reduced thermal noise magnitude of 0.1, 1 and 5, respectively. The color bar denotes the angle between the polar vectors and the [100] direction. Temperature is 350 K.

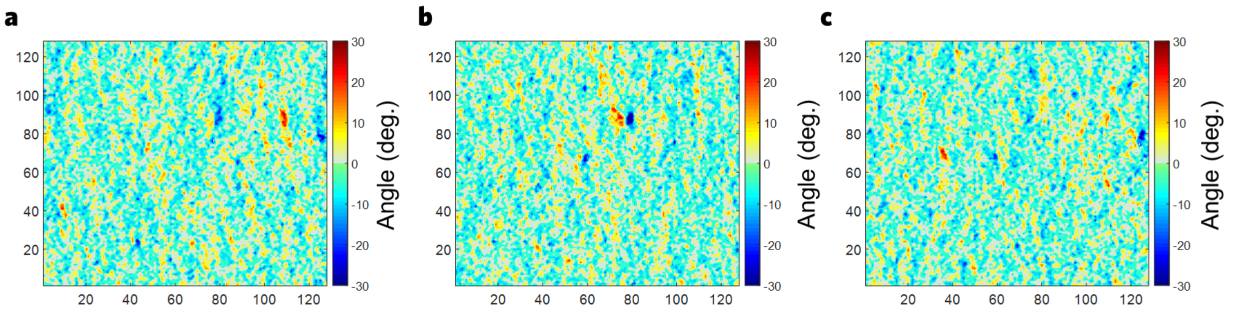

Supplementary Figure 23. Simulated domain morphology for a [100]-poled PNR-ferroelectric composite at 350 K with the reduced thermal noise magnitude of 5. **a**, **b** and **c** are obtained at different time step, being 1000, 2000 and 3000, respectively. The color bar denotes the angle between the polar vectors and the [100] direction.

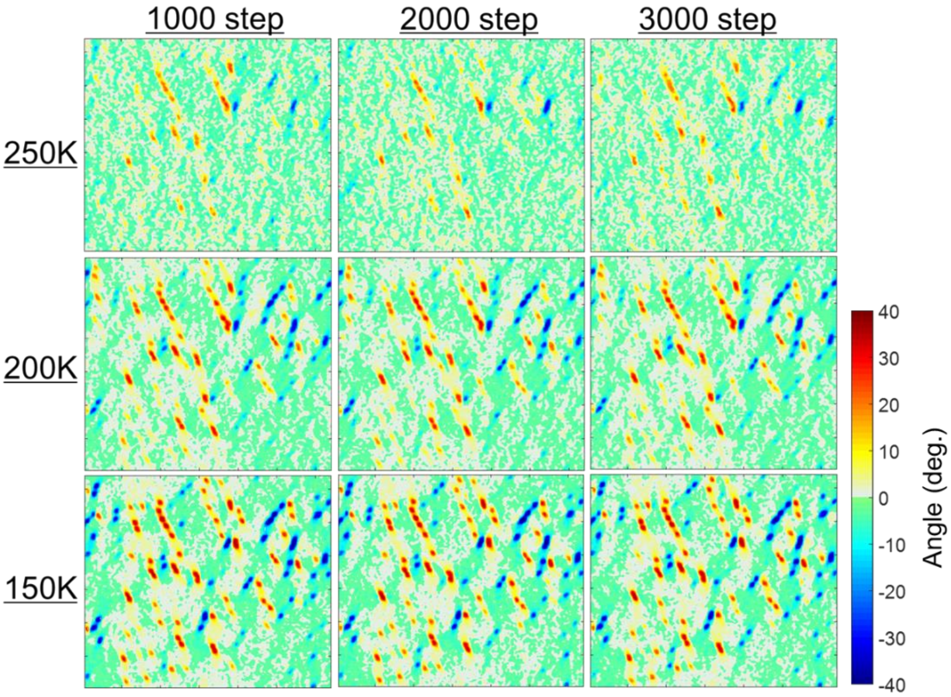

Supplementary Figure 24. Simulated domain morphology for a [100]-poled PNR-ferroelectric composite at 150 K, 200 K and 250 K with the reduced thermal noise magnitude of 5. The microstructures are shown at different time steps, being the 1000, 2000 and 3000. The color bar denotes the angle between the polar vectors and the [100] direction.

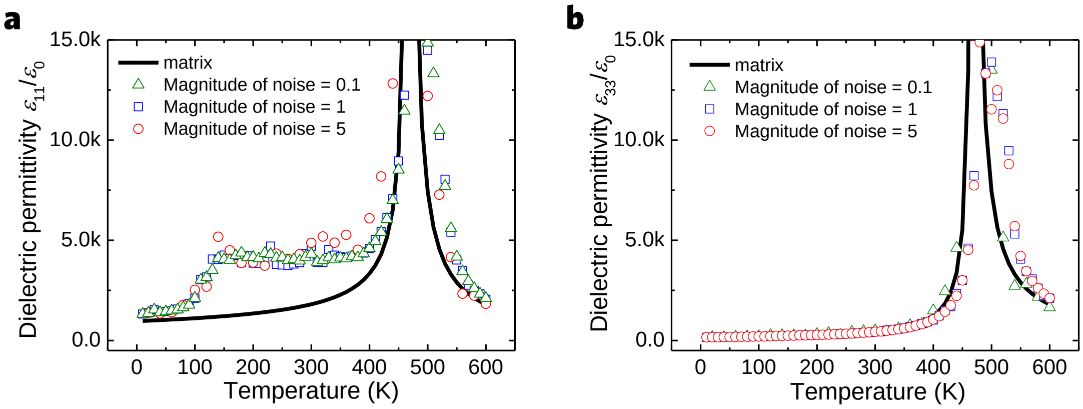

Supplementary Figure 25. Simulated temperature dependence of dielectric permittivities for PNR-ferroelectric composites under different levels of thermal noise. **a**, the transverse dielectric permittivity $\varepsilon_{11}/\varepsilon_0$; **b**, the longitudinal dielectric permittivity $\varepsilon_{33}/\varepsilon_0$.

Supplementary Table 1. Dielectric and piezoelectric coefficients of relaxor-PT and PZT crystals. The data for PZT crystals is from the phenomenological calculation.

Relaxor crystals	Data for single domain state			
	$\varepsilon_{33}/\varepsilon_0$	$\varepsilon_{11}/\varepsilon_0$	d_{33} (pC N^{-1})	d_{15} (pC N^{-1})
PMN-0.28PT(R)*	750	5200	90	2900
PMN-0.32PT (O)*	800	6000 ($\varepsilon_{22}/\varepsilon_0$~22000)	200	3600 (d_{24} ~2200)
PZN-0.08PT (R) [1]	700	11000	90	6000
PZN-0.15PT (T)*	600	5500	380	900
PMN-0.12PT (T) *	870	12000	520	1500
$Pb(Zr_{0.52}Ti_{0.48})O_3$(R)[2-3]	450	1200	130	1350
$Pb(Zr_{0.48}Ti_{0.52})O_3$(T)[2-3]	350	1500	300	500

* data obtained in this work.

Supplementary Table 2. Refined structural parameters for the rhombohedral PMN-0.28PT solid solution. The Rietveld refinement was carried out using TOPAS Academic[4], and the anisotropic strain broadening function proposed by Stephens[5], was incorporated into the refinements. It can be seen that the long-range structure of PMN-0.28PT is *R3m* over the temperature range of 100-300K.

	Temp. [K]	100	125	150	200	250	300
R3m	*a* [Å]	4.02090(1)	4.02111(1)	4.02124(1)	4.02159(1)	4.02194(1)	4.02239(1)
	alpha	89.8228(7)	89.8284(7)	89.8352(7)	89.8513(7)	89.8716(7)	89.8942(5)
	Δ(Pb)	0.0270(3)	0.0262(2)	0.0260(3)	0.0254(3)	0.0224(4)	0.0209(5)
	Δ(Nb/Mg/Ti)	0.0146(3)	0.0142(3)	0.0143(3)	0.0139(4)	0.0122(5)	0.0111(6)
	R_{wp} [%]	11.16	11.17	11.26	11.39	11.33	11.29
	R_{bragg} [%]	1.72	1.56	1.82	2.02	2.02	1.96

Supplementary Note 1: Analysis of frequency-dependent transverse dielectric responses for relaxor-PT crystals.

The frequency-dependent dielectric permittivity and loss of PMN-0.32PT and PZN-0.15PT crystals are shown in Supplementary Figure 8. In the frequency range of 10^{-1}-10^{5} Hz, frequency dependent behaviors were clearly observed at the temperature below 200 K.

According to Debye theory, the relaxational dielectric spectrum can be described by means of the distribution function of the relaxation time g(τ)[6]:

$$\varepsilon(\omega) = \varepsilon_\infty + \Delta\varepsilon \int_0^\infty \frac{g(\tau)}{1+\omega^2\tau^2} d\ln(\tau) \qquad (1)$$

where g(τ) is normalized by the following equation:

$$\int_0^\infty g(\tau) d\ln(\tau) = 1 \qquad (2)$$

In this study, we assume there is a strong Debye relaxation with a constant distribution between certain upper value (τ_1) and lower value (τ_2). Thus, the $g(\tau)$ is assumed to be:

$$g(\tau) = \begin{cases} 1/\ln(\tau_1/\tau_2) & \tau_2 \le \tau \le \tau_1 \\ 0 & \tau > \tau_1, \tau < \tau_2 \end{cases} \qquad (3)$$

In this condition, ε_∞ represents the dielectric permittivity at frequency much higher than $1/\tau_2$. $\Delta\varepsilon$ represents the contribution of the relaxation to the static permittivity. Based on Eqs. 1-3, the frequency dependent dielectric permittivity can be expressed as:

$$\varepsilon(\omega) = \varepsilon_\infty + \frac{\Delta\varepsilon}{\ln(\tau_1/\tau_2)}[\ln(\tau_1/\tau_2) - \frac{1}{2}\ln(\frac{1+\omega^2\tau_1^2}{1+\omega^2\tau_2^2})] \qquad (4)$$

The dielectric spectra of PMN-0.32PT and PZN-0.15PT crystals are fitted by using Eq. 4 and given in Supplementary Figure 9. It can be seen that the upper frequency limit is around 10^{7}-10^{8} for PZN-0.15PT and PMN-0.32PT, being much lower than the frequency of phonon modes (10^{12}-10^{14} Hz). The observed relaxation frequency range (10^{0}-10^{8}) is similar to that of dipole reorientation. This indicates that some interface motion or dipoles' switching significantly contributes to the dielectric response at 120 K and 150 K for relaxor-PT crystals.

Supplementary Note 2: Inference of the size of PNRs by Arrhenius law.

Supplementary Figure 10 a, b and c show the temperature dependence of the imaginary part ($\varepsilon''/\varepsilon_0$) of the transverse dielectric permittivity for PMN-0.28PT, PMN-0.32PT and PZN-0.15PT crystals, respectively. Supplementary Figure 10 d, e and f show the temperature of maximum $\varepsilon''/\varepsilon_0$ versus frequency. It can be seen that ln(f) and $1/T_m$ follow a linear relationship, which can be expressed by Arrhenius law:

$$f = f_0 \exp[-E_a/(k_B T_m)] \tag{5}$$

where f is the measurement frequency, E_a the activation energy, k_B the Boltzmann constant, f_0 the attempt frequency, and T_m the temperature of maximum $\varepsilon''/\varepsilon_0$. By fitting Eq. 5, it is found that the activation energy E_a is around $2000k_B$ for PMN-0.28PT, PMN-0.32PT and PZN-0.15PT crystals. Activation energy E_a can be expressed as ΔGV, where ΔG is the energy barrier density for reorientation of a PNR and V the volume of a single PNR. According to Landau-Devonshire formalism of free energy [2,7], the typical values of ΔG are in the range of 10^5~10^7 J m^{-3}. Based on this typical value of ΔG, the volume of a PNR (V) is calculated to be in the range of $(27.4\sim274)\times10^{-27}$ m^3. Thus, the size of PNRs ($\sqrt[3]{V}$) is inferred to be in the range of 1.4~6.5 nm.

Supplementary Note 3: Framework and numerical simulation of the phase-field model

(1) Framework of the phase field model

The temporal evolution of a polarization field is described by the Time-Dependent Ginzburg-Landau (TDGL) equation,

$$\frac{\partial P_i(\mathbf{r},t)}{\partial t} = -L\frac{\delta F}{\delta P_i(\mathbf{r},t)} + \xi_i(\mathbf{r},t), \quad (i=1,2,3), \tag{6}$$

where L is the kinetic coefficient, F, the total free energy of the system, and $P_i(\mathbf{r},t)$ is the polarization. $\delta F/\delta P_i(\mathbf{r},t)$ is the thermal dynamic driving force for the spatial and temporal evolution of $\partial P_i(\mathbf{r},t)$. $\xi_i(\mathbf{r},t)$ represents the effect of thermal noise, which is a Gaussian random fluctuation satisfying:

$$<\xi_i(\mathbf{r},t)>=0 \quad \text{and} \quad <\xi_i(\mathbf{r},t)\xi_j(\mathbf{r}',t')>=Tm\delta(\mathbf{r}-\mathbf{r}')\delta(t-t') \tag{7}$$

where T is the temperature, m represents the amplitude of thermal noise, δ_{ij} is the Kronecker delta, $\delta(\mathbf{r}-\mathbf{r}')$ and $\delta(t-t')$ are the delta function. The bracket <…> denotes an average value.

The total free energy of the system includes the bulk free energy, elastic energy, electrostatic energy, and the gradient energy:

$$F = \int_V [f_{\text{bulk}} + f_{\text{elas}} + f_{\text{elec}} + f_{\text{grad}}]dV \tag{8}$$

where V is the system volume of the PNR-ferroelectric composite, f_{bulk} denotes the Landau bulk free energy density, f_{elas} the elastic energy density, f_{elec} the electrostatic energy density and f_{grad} the gradient energy density.

The bulk free energy density is expressed by Landau theory, i.e.

$$\begin{aligned} f_{\text{bulk}} = {} & \alpha_1(P_1^2+P_2^2+P_3^2)+\alpha_{11}(P_1^4+P_2^4+P_3^4)+\alpha_{12}(P_1^2P_2^2+P_2^2P_3^2+P_3^2P_1^2) \\ & +\alpha_{111}(P_1^6+P_2^6+P_3^6)+\alpha_{112}[P_1^4(P_2^2+P_3^2)+P_2^4(P_1^2+P_3^2)+P_3^4(P_1^2+P_2^2)] \\ & +\alpha_{123}P_1^2P_2^2P_3^2 \end{aligned} \tag{9}$$

where α_1, α_{11}, α_{12}, α_{111}, α_{112} and α_{123} are Landau energy coefficients. The values of these coefficients determine the thermodynamic behaviors of the bulk phases (paraelectric or ferroelectric). In our simulation work, the difference between the ferroelectric matrix and PNRs are reflected in the f_{bulk}, where different Landau coefficients were used for matrix and PNRs, respectively.

The gradient energy density, associated with the formation and evolution of domain walls, can be expressed as:

$$\begin{aligned} f_{\text{grad}} &= \frac{1}{2}G_{11}(P_{1,1}^2+P_{2,2}^2+P_{3,3}^2)+G_{12}(P_{1,1}P_{2,2}+P_{2,2}P_{3,3}+P_{1,1}P_{3,3}) \\ &+\frac{1}{2}G_{44}[(P_{1,2}+P_{2,1})^2+(P_{2,3}+P_{3,2})^2+(P_{1,3}+P_{3,1})^2] \\ &+\frac{1}{2}G_{44}^{'}[(P_{1,2}-P_{2,1})^2+(P_{2,3}-P_{3,2})^2+(P_{1,3}-P_{3,1})^2] \end{aligned} \tag{10}$$

where G_{ij} are gradient energy coefficients. $P_{i,j}$ denote $\partial P_i/\partial r_j$.

The corresponding elastic energy density can be expressed as:

$$f_{\text{elas}} = \frac{1}{2}c_{ijkl}e_{ij}e_{kl} = \frac{1}{2}c_{ijkl}(\varepsilon_{ij}-\varepsilon_{ij}^0)(\varepsilon_{kl}-\varepsilon_{kl}^0) \tag{11}$$

where c_{ijkl} is the elastic stiffness tensor, ε_{ij} the total strain, ε_{kl}^0 the electrostrictive stress-free strain, i.e., $\varepsilon_{kl}^0 = Q_{ijkl}P_kP_l$.

The electrostatic energy density is given by:

$$f_{\text{elec}} = -\frac{1}{2}E_i^{in}P_i - E_i^{ex}P_i \tag{12}$$

where E_i^{in} is the E-field induced by the dipole moments in the specimen. The detailed expression of E_i^{in} can be found in Ref. 8. E_i^{ex} is an applied external E-field.

(2) Material constants and numerical simulation parameters of PNR-ferroelectric composite

A semi-implicit Fourier-spectral method is adopted for numerically solving the TDGL equation[9]. Based on the experimental data (polarization and dielectric response) and the parameters of typical perovskite ferroelectrics ($Pb(Zr,Ti)O_3$ and $BaTiO_3$)[2,10], the following parameters are adopted in the present simulation. For the ferroelectric matrix, the Landau free energy parameters are: $\alpha_1 = 2.38(T-468)\times10^5 C^{-2}m^2N$, $\alpha_{11} = 5.56\times10^7 C^{-4}m^6N$, $\alpha_{12} = 3.37\times10^8 C^{-4}m^6N$, $\alpha_{111} = 3.23\times10^8 C^{-6}m^{10}N$, $\alpha_{112} = 1.02\times10^9 C^{-6}m^{10}N$, $\alpha_{123} = -1.21\times10^9 C^{-6}m^{10}N$. In the whole temperature range, there is only a Cubic-Tetragonal phase transition at 468 K for the ferroelectric matrix. The spontaneous polarization of matrix is about 0.39 C m^{-2} at 300 K, being similar to the experimental value of the PZN-0.15PT crystal. At 20 K, the dielectric permittivity $\varepsilon_{11}/\varepsilon_0$ and $\varepsilon_{33}/\varepsilon_0$ are 850 and 160 respectively for ferroelectric matrix, being similar to the measured data of PZN-0.15PT (At temperature of 20 K and frequency of 100 Hz, the measured $\varepsilon_{11}/\varepsilon_0$ and $\varepsilon_{33}/\varepsilon_0$ are 930 and 250, respectively.) For PNRs, the Landau free energy parameters are: $\alpha_1 = 2.2(T-620)\times10^5 C^{-2}m^2N$, $\alpha_{11} = -2.67\times10^8 C^{-4}m^6N$, $\alpha_{12} = 2.67\times10^8 C^{-4}m^6N$, $\alpha_{111} = 3.33\times10^9 C^{-6}m^{10}N$, $\alpha_{112} = 3.33\times10^8 C^{-6}m^{10}N$, $\alpha_{123} = 3.0\times10^{10} C^{-6}m^{10}N$, where T is the temperature in Kelvin. In the whole temperature range, there is only a Cubic-orthorhombic phase transition around 620 K for PNRs. The spontaneous polarization of PNR is 0.41 C m^{-2} at 300 K without consideration of interactions from the matrix. The elastic constants and electrostrictive coefficients are set to be the same for the matrix

and PNRs: $s_{11}^D = 20\times10^{-12} m^2N^{-1}$, $s_{12}^D = -7.5\times10^{-12} m^2N^{-1}$, $s_{44}^D = 20\times10^{-12} m^2N^{-1}$, $Q_{11} = 0.089C^{-2}m^4$, $Q_{12} = -0.030C^{-2}m^4, Q_{44} = 0.034C^{-2}m^4$ (the elastic constants refer to the data of PZN-0.12PT crystal[11] and the electrostrictive coefficients refer to data for $PbTiO_3$ [2]). The background dielectric permittivity (k_b), associated with the contribution of the hard mode to the permittivity[12], is set to be 100×ε_0. In the computer simulations, we employed 2D 128×128 discrete grid points (3D: 64×64×64 grid points) and periodic boundary conditions. The grid space in real space is chosen to be $\Delta x = \Delta y = \Delta z = 1nm$. The gradient energy coefficients are chosen to be $G_{11}/G_{110} = 1.5$, $G_{12}/G_{110} = 0$, $G_{44}/G_{110} = G_{44}/G_{110} = 0.75$, where $G_{110} = 7.04\times10^{-11}C^{-2}m^4N$ [13]. Based on these parameters, the simulated width of domain walls was found to be 1-2 nm, being consistent with existing experimental measurements[14,15]. The time step for integration is $\Delta t/t_0 = 0.01$, where $t_0 = 1/L\alpha_1|_{T=300K}$. For 2D simulation, P_z is set at zero.

Supplementary Note 4: The effect of PNRs volume fraction on the transverse dielectric response for the PNR-ferroelectric composite.

The temperature dependent dielectric permittivities were simulated under different PNR volume fractions for a PNR-ferroelectric composite, as shown in Supplementary Figure 12. It was found that the increase of volume fraction of PNRs may bring two effects. First, higher volume fraction of PNRs may result in higher dielectric response when most of the PNRs are unstable or in "collinear" state. Secondly, higher volume fraction of PNRs could result in stronger interaction among PNRs due to the decrease of average distances among PNRs. Thus, PNRs are more stable for higher volume fractions, where higher temperatures are needed to make PNRs unstable and/or in "collinear" state. As shown in Supplementary Figure 12, a composite with higher volume fraction of PNRs shows higher dielectric response at elevated temperature region, but at the cost of temperature stability.

Supplementary Note 5: The effect of thermal fluctuations on the dielectric responses for the PNR-ferroelectric composite.

Thermal fluctuation is considered in phase-field simulation, where it is randomly changed for every step. The magnitude of thermal noise is proportional to the temperature, as given in Eq. 7. Supplementary Figure 22 exhibits the impact of thermal noise on the polarization of the tetragonal matrix at 350 K. The maximum angle between polar vector and [100] direction is about 7° and 20° for the reduced thermal noise magnitude of 1 and 5, respectively. Obviously, the thermal noise with magnitude of 5 is extremely large, which will be used to check the extreme condition in our simulations.

Supplementary Figure 23 shows the microstructure of PNR-ferroelectric composite at 350 K with thermal noise magnitude of 5. Due to the impact of thermal noise, the polar vector for specific grids varied at different time. The maximum angle between polar vector and [100] direction is about 30°. The maximum angle generally comes from the "collinear" PNRs, since they are easy to be rotated compared to the tetragonal matrix. Supplementary Figure 24 shows microstructure of a PNR-ferroelectric composite at 150-250 K with reduced thermal noise magnitude of 5. It can be seen that the PNRs are not affected too much by thermal fluctuation at different time steps.

Supplementary Figure 25 shows the calculated temperature-dependent dielectric permittivities for

PNR-ferroelectric composite at different levels of thermal fluctuation. Over all, the temperature dependent properties aren’t affected significantly by thermal noise, though a bit higher fluctuation is observed for the magnitude of 5.

Supplementary references

1. Jin, J., Rajan, K. K. & Lim, L.C. Properties of single domain Pb($Zn_{1/3}Nb_{2/3}$)O_3-(6-7)%$PbTiO_3$ single crystal. *Jpn. J. Appl. Phys.* **45**, 8744-8747 (2006).
2. Haun, M. J., Furman, E. S., Jang, J. &. Cross L. E. Thermodynamic theory of the lead zirconate-titanate solid-solution system, part I-V: Theoretical calculations. *Ferroelectrics* **99**, 13-86 (1989).
3. Du, X. H., Zheng, J., Belegundu, U., & Uchino, K. Crystal orientation dependence of piezoelectric properties of lead zirconate titanate near the morphotropic phase boundary. *Appl. Phys. Let.* **72**, 2421-2423 (1998).
4. Coelho, A. A. A bound constrained conjugate gradient solution method as applied to crystallographic refinement problems. *J. Appl. Cryst.* **38**, 455-461 (2005).
5. Stephens, P. W. Phenomenological model of anisotropic peak broadening in powder diffraction. *J. Appl. Cryst.* **32**, 281-289 (1999).
6. Jonscher, A. K. *Dielectric Relaxation in Solids* (London: Chelsea Dielectric, 1983).
7. Bell, A. J. Calculations of dielectric properties from the superparaelectric model of relaxors. *J. Phys.: Condens. Matter* **5**, 8773-8792 (1993).
8. Hu, H. L. & Chen, L. Q. Three-dimensional computer simulation of ferroelectric domain formation. *J. Am. Ceram. Soc.* **81**, 492-500 (1998).
9. Chen, L. Q. & Shen J. Applications of semi-implicit Fourier-spectral method to phase field equations. *Comput. Phys. Commun.* **108**, 147-158 (1998).
10. Chen, L. Q. *APPENDIX A–Landau free-energy coefficients.* In Physics of Ferroelectrics (pp. 363-372). Springer Berlin Heidelberg (2007).
11. Li, F., *et al*. Electromechanical properties of tetragonal Pb ($In_{1/2}Nb_{1/2}$)O_3–Pb ($Mg_{1/3}Nb_{2/3}$) O_3–$PbTiO_3$ ferroelectric crystals. *J. Appl. Phys.* **107**, 054107 (2010).
12. Tagantsev, A. K. Landau expansion for ferroelectrics: which variable to use? *Ferroelectrics* **375**, 19-27 (2008).
13. Li, Y. L., Hu, S. Y., Liu, Z. K., & Chen, L. Q. Effect of substrate constraint on the stability and evolution of ferroelectric domain structures in thin films. *Acta Mater.* **50**, 395-411 (2002).
14. Jia, C. L., Mi, S. B., Urban, K., Vrejoiu, I., Alexe, M., & Hesse, D. Atomic-scale study of electric dipoles near charged and uncharged domain walls in ferroelectric films. *Nat. Mater.* **7**, 57-61 (2008).
15. Stemmer, S., Streiffer, S. K., Ernst, F., & Rüuhle, M. Atomistic structure of 90° domain walls in ferroelectric $PbTiO_3$ thin films. *Phil. Maga. A* **71**, 713-724 (1995).
