# Supplementary figures and images for: The origin of ultrahigh piezoelectricity in relaxor-ferroelectric solid solution crystals
Source: Nat Commun. 2016 Dec 19;7:13807. doi: 10.1038/ncomms13807 (PMC5187463; doi:10.1038/ncomms13807)

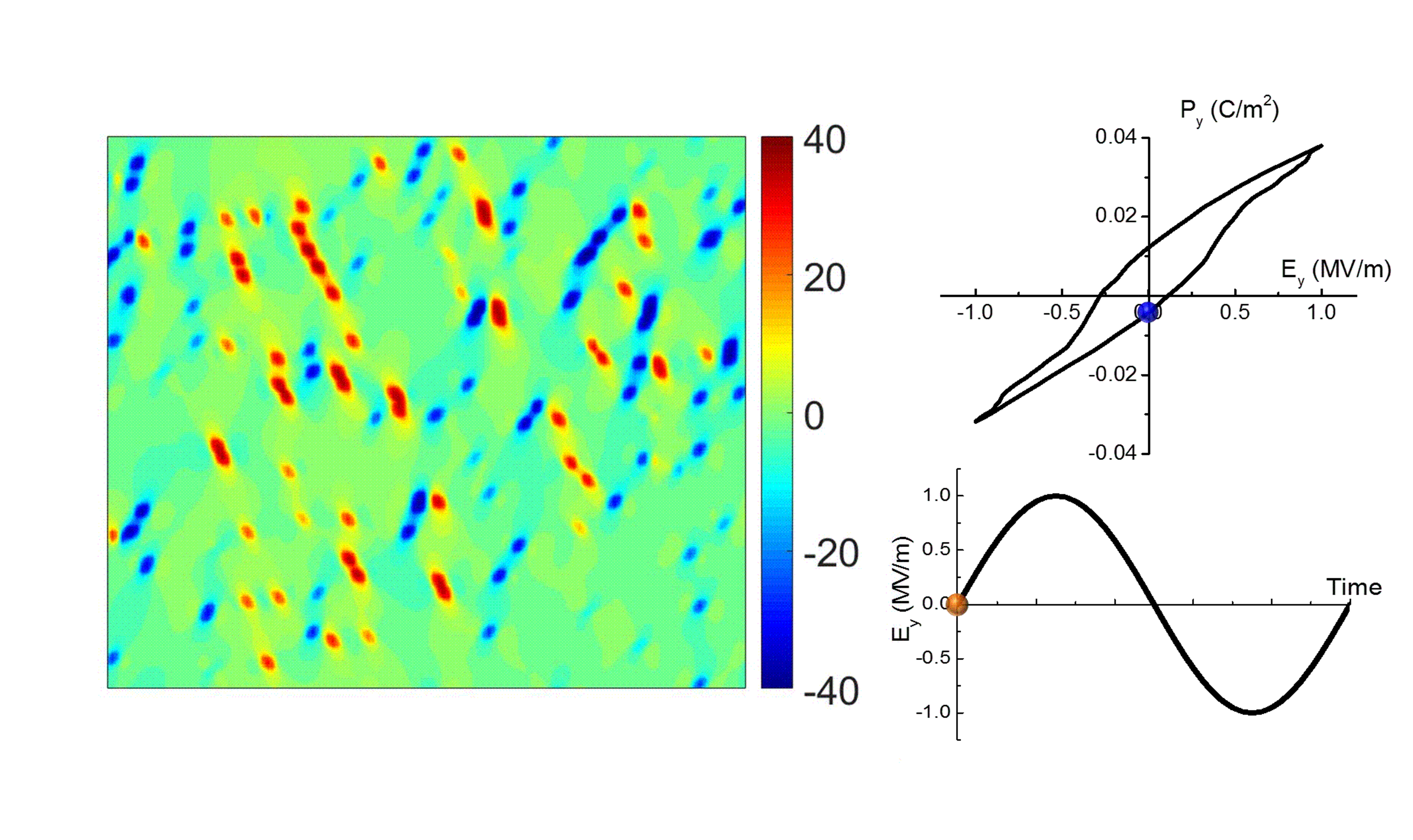

Supplement: Supplementary Movie 1 — Perpendicular [010] E-field induced microstructural variation for a [100]-poled PNR-ferroelectric composite at 150 K. [file ncomms13807-s3.tif]

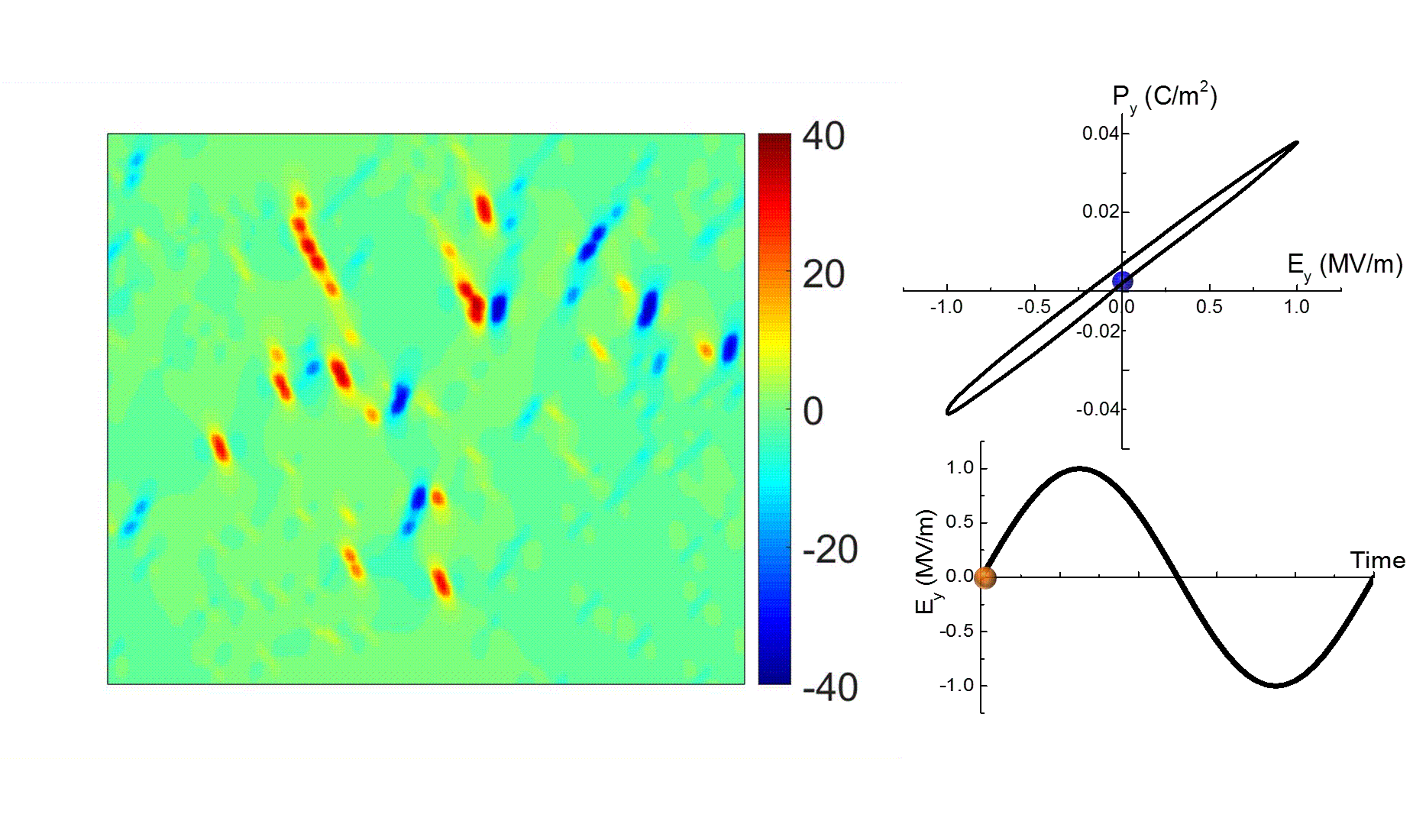

Supplement: Supplementary Movie 2 — Perpendicular [010] E-field induced microstructural variation for a [100]-poled PNR-ferroelectric composite at 250 K. [file ncomms13807-s4.tif]

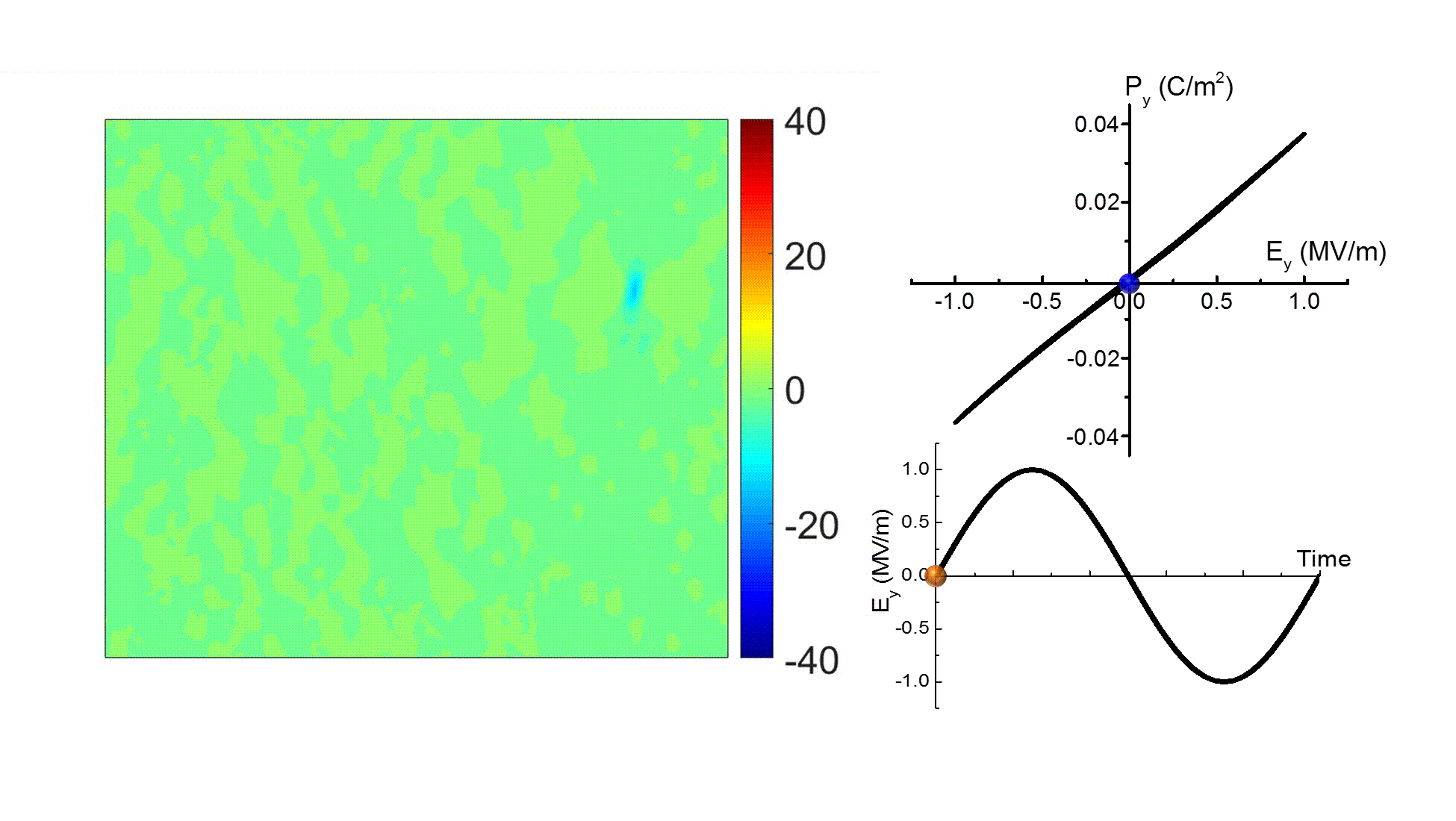

Supplement: Supplementary Movie 3 — Perpendicular [010] E-field induced microstructural variation for a [100]-poled PNR-ferroelectric composite at 350 K. [file ncomms13807-s5.tif]
